# Aggregation mechanisms for crowd predictions

**DOI:** 10.1007/s10683-019-09631-0

**Published:** 2019-11-09

**Authors:** Stefan Palan, Jürgen Huber, Larissa Senninger

**Affiliations:** 1 Department of Banking and Finance, University of Graz, Universitätsstraße 15, 8010 Graz, Austria; 2 Department of Banking and Finance, University of Innsbruck, Universitätsstraße 15, 6020 Innsbruck, Austria

**Keywords:** Information aggregation, Asymmetric information, Wisdom of crowds, C53, C83, G14

## Abstract

When the information of many individuals is pooled, the resulting aggregate often is a good predictor of unknown quantities or facts. This aggregate predictor frequently outperforms the forecasts of experts or even the best individual forecast included in the aggregation process (“wisdom of crowds”). However, an appropriate aggregation mechanism is considered crucial to reaping the benefits of a “wise crowd”. Of the many possible ways to aggregate individual forecasts, we compare (uncensored and censored) arithmetic and geometric mean and median, continuous double auction market prices and sealed bid-offer call market prices in a controlled experiment. We use an asymmetric information structure, where participants know different sub-sets of the total information needed to exactly calculate the asset value to be estimated. We find that prices from continuous double auction markets clearly outperform all alternative approaches for aggregating dispersed information and that information lets only the best-informed participants generate excess returns.

**Electronic supplementary material:**

The online version of this article (10.1007/s10683-019-09631-0) contains supplementary material, which is available to authorized users.

## Introduction

### Motivation

“Wisdom of crowds”, after Surowiecki’s (2004) book of the same name, is a term used to describe the observation that the aggregate of forecasts by multiple people is often a better predictor of actual outcomes than the forecasts of experts or even the best individual forecast included in the aggregation process. A number of studies have set out to document this outperformance (e.g., Gordon 1924; Bruce 1935; Sauer 1998; Berg et al. 2008a, b) and to explore and describe which forecasters and forecasting targets most readily lend themselves to successful crowd prediction (e.g., Lorge and Fox 1958; Brown and Sauer 1993; Berg and Rietz 2003; Gruca et al. 2003; Polgreen et al. 2007; Davis-Stober et al. 2014).

In the present paper, we aim to compare different mechanisms for aggregating crowd predictions in a setting with asymmetric information. We are particularly interested in the predictive accuracy resulting from these aggregation mechanisms. Our experiment includes very simple mechanisms, like the average or median of individual predictions, and more complex ones, like the prices from a continuous double auction market. We aim to answer the question whether simple mechanisms perform equally well or even better than more complex ones and should thus be the instruments of choice, or whether more complex mechanisms yield better predictions, which offset their higher costs in terms of time and infrastructure expenditures. We are of course not the first to ask this question. In work directly related to ours, Clemen (1989) provides a review of the literature on combining forecasts. He reports that in the majority of cases simple aggregation mechanisms are more effective than more complex ones. This result is supported by the more recent work of Soll et al. (2009), who report that simple averaging is the most effective way of combining individual judgments. Other findings in favor of averaging individual estimates are Budescu and Yu (2006) and Lichtendahl Jr et al. (2013) (both comparing it to using Bayes’ rule), and Mannes et al. (2012) (in effect comparing the average to randomly choosing an individual estimate).^1^ One more sophisticated averaging approach is advanced by Budescu and Chen (2014), who use a model to identify experts in the crowd and weight their opinions by relevance before aggregating the individual estimates to a group opinion.

In a more nuanced finding, Malone et al. (2009) argue that averaging is a surprisingly good tool when estimating a certain number, but that in more complex situations more complex mechanisms are needed to aggregate information efficiently. They list “prediction markets” and other markets with monetary or non-monetary incentives as being such mechanisms. In line with this view, market-based mechanisms have gained significant attention in recent decades. In prediction markets, the market’s organizers create an asset whose value is tied to the outcome to be estimated.^2^ Defining such assets thus transforms the estimation of an unknown outcome or of its probability into a task that can be accomplished by a market. In markets, prices have the dual role of (1) aggregating existing, distributed information, and of (2) inducing traders to seek out new information. There is an extensive literature on (1), including such important contributions as Diamond and Verrecchia (1981), Plott and Sunder (1988), Forsythe and Lundholm (1990), Ostrovsky (2012), and Choo et al. (2019). We see our own paper as contributing to this literature. Nevertheless, in addition to aggregating existing information, the existence of markets can also lead to the search for (or possibly even the creation of) new information. In order to prevent confounding effects, we rule this out in our experiment.

The main innovation in our paper lies in combining a design featuring asymmetric, cumulative information with different aggregation mechanisms. While these or similar aggregation mechanisms have been explored before (see the references above), asymmetric information remains underexplored: if considered at all, the information is usually partitioned into *no information* versus *full information*, or, to use a metaphor from capital markets, noise traders versus corporate insiders. In this paper, we simplify by abstracting from the near-continuum of different information levels and information sets found outside of the lab, but extend the typical analysis approach to four different information levels, interacted with different aggregation mechanisms. We are thus able to derive findings that could not have been obtained with only one or two information levels—e.g., that additional (correct) information is not always helpful, but rather profits only the best-informed agents. This connects nicely to the literature from the field (see e.g, Schredelseker 1984; Huber 2007; Huber et al. 2008), and extends this finding to encompass the different aggregation mechanisms we explore.

We study a setting with asymmetric information, since in many relevant prediction environments (e.g., future stock prices, betting outcomes, etc.), different participants will typically have different information and—even more relevant—information of different quality. We mimic this with our experimental design, which allows us to establish better and worse informed participants, as the information levels are cumulative. This means that a higher information level includes all of the information of the levels below plus additional relevant information that the lower levels lack. Specifically, we let participants estimate the value of coins contained in jars, each of which every participant is allowed to inspect for 15 s. Participants then receive different levels of explicit information on the number of different types of coins in the jars. With this design we explore if, to whom, and to which degree additional information is useful in such an aggregation task. Specifically, we aim to find answers to the hypotheses developed in the following section.

### Hypotheses

The existing literature, among others Sunder (1992) and Huber (2007), leads us to expect that having more of some relevant information will be useful in making more precise estimates in an estimation task. So, while the null of our first hypothesis would be to find no differences in estimation errors when comparing the four different information levels in our experiment, the alternative hypothesis is that the higher the information level, the lower will be the estimation error.

#### Hypothesis 1

The higher the information level, the lower the estimation error.

We design our experiment to also test the effect of experience. We accomplish this by repeating the same estimation task three times, with trading in a market setting interposed between the three instances of estimate elicitation. In other words, the value of the same jar needs to be estimated in three consecutive trading periods, allowing our participants to learn from their observations in the market for jars. While the null hypothesis regarding these learning effects is that estimation errors are equal in periods 1, 2, and 3 of trading a given jar, the alternative is that estimation errors will be lower in later periods.

#### Hypothesis 2

Estimation error decreases over consecutive periods of trading the same jar.

We next turn to trading profits and consider whether participants’ payoffs will depend on their performance as estimators. The null hypothesis would be that traders’ profits will be equal despite differences in how close these traders’ estimates are to true jar values. For the alternative hypothesis, we expect that participants with smaller estimation errors (irrespective of information level) should be able to generate greater returns.

#### Hypothesis 3

The smaller the absolute deviation of traders’ estimates from true jar values, the greater participants’ final wealth.

Furthermore, the literature (e.g., Schredelseker 1984; Huber 2007; Huber et al. 2008) leads us to expect that better-informed participants, especially the best-informed, will be able to generate trading profits and thus earn excess returns. Hence, while the null hypothesis postulates that the final wealth of all participants will be identical, the alternative is that better-informed traders will have greater final wealth.

#### Hypothesis 4

The higher the information level, the higher participants’ final wealth.

Finally, regarding the comparison between aggregation mechanisms, we expect that in the fairly complex estimation task we employ, and with repetition, aggregation mechanisms with opportunities for learning (such as the continuous double auction) will perform better than mechanisms without such opportunities. Furthermore, we expect mechanisms (such as the two market mechanisms) where participants can express their participative confidence in their own forecasts monetarily to outperform the simpler mechanisms, where each estimate is weighted equally. So while the null hypothesis for capturing this idea would be that there is no difference in the estimation error across the different aggregation mechanisms, the alternative is that the continuous double auction will have the lowest estimation error (due to it offering the greatest learning opportunities), followed by the call-auction (which offers fewer learning opportunities, but also allows traders to “put their money where their mouth is”), and followed by means and medians, which will have the highest estimation errors (without differences between different versions of these simpler aggregation mechanisms).

#### Hypothesis 5

Forecast error will be lowest when aggregating using the continuous double auction, followed by the call auction, followed by means and median (with no difference between the latter).

## Experimental design

We propose a research design which is simple, easy to understand, and allows studying our research question under controlled conditions. Using a laboratory experiment, we first let participants estimate the value of a jar filled with coins. We then provide them with partial information about the coins in the jar and elicit updated estimates. Finally, participants trade the jars in a market, which aggregates their dispersed and noisy information into market prices. This procedure allows us to analyze—and compare the performance of—multiple mechanisms for aggregating dispersed information. The mechanisms we study are (1) (censored) means and medians of individual, incentivized estimates, (2) mean, median and closing prices as well as the closing bid-ask midpoint of a continuous double auction, and (3) the uniform settlement price from a sealed bid-ask call auction.

### Assets and information levels

**Fig. 1:**
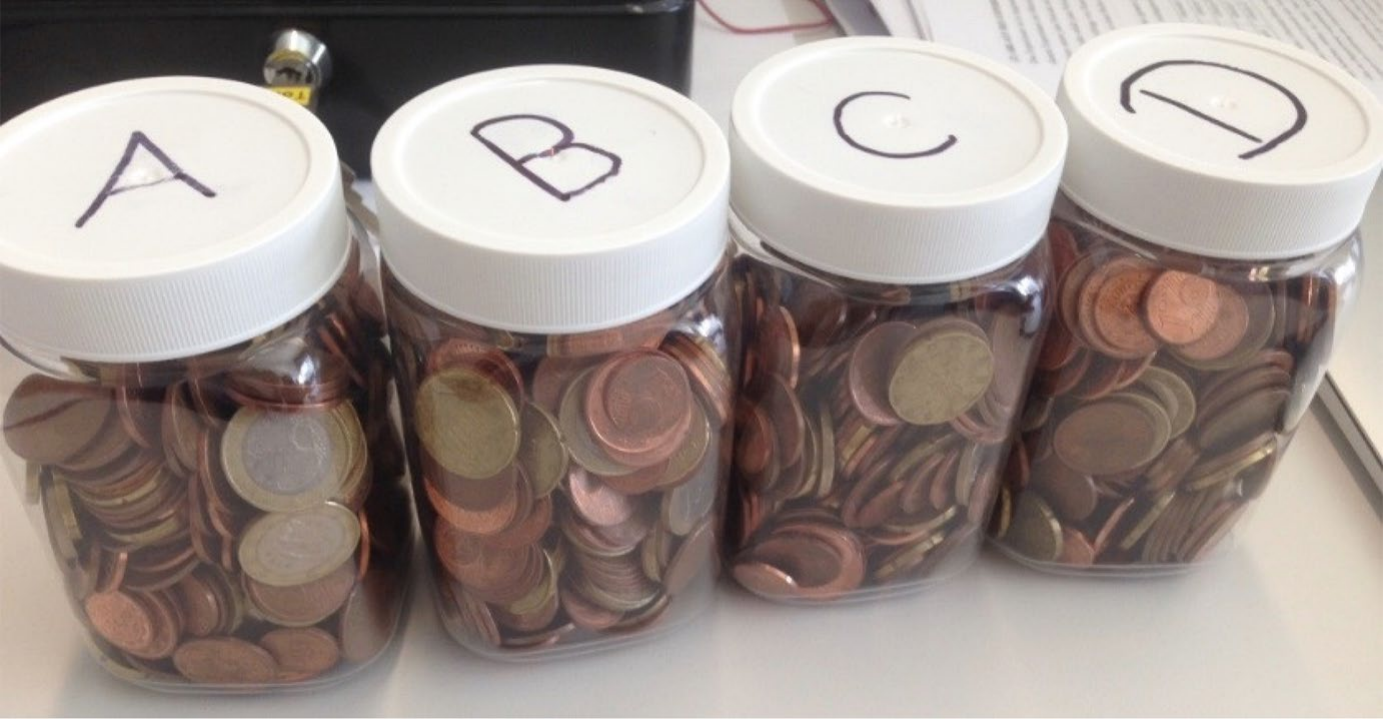
Photo of the four plastic jars employed in the experiment

In preparation for our experiment, we fill four plastic jars with 1-euro and 20-, 5- and 1-cent coins. Figure 1 shows a photo and Table 1 presents information about the value of the coins in each of the jars, designated A through D.

**Table 1:** Value of coins in jars

Jar	A	B	C	D	Total
1 euro	9	10	7	6	32
20 cents	7.2	9.6	8.4	6.8	32
5 cents	7.05	9	7.6	8.35	32
1 cent	1.26	0.66	0.88	1.2	4
Total	24.51	29.26	23.88	22.35	100

Jars contain an average of 25 euros (s.d. 2.58), made up of, on average, 8 euros each in coins of 1 euro (s.d. 1.58 euros), 20 cents (s.d. 1.10 euros), and 5 cents (s.d. 0.74 euros) as well as 1 euro in coins of 1 cent (s.d. 0.24 euros). Participants are informed that these four types of coins are contained in each of the jars. They can also obtain (imperfect) information about the value of the coins contained in each individual jar^3^ from two sources. First, each participant is handed each of the jars for 15 s to view, turn, weigh in their hands, etc. Participants are not allowed to open the jars or use any means other than their senses to analyze the jars’ contents. Second, participants are provided with one of four information levels for each of the jars. More precisely, each participant receives information level I0 for one of the jars, I1 for another jar, I2 for yet another and I3 for the fourth jar. Participants assigned information level I0 do not receive any additional information about the coins in the jar. Participants assigned level I1 receive full information about the number (and, separately stated on the computer screen, value) of the 1-euro coins in the jar. Participants assigned level I2 receive full information about the number (and value) of the 20-cent coins in the jar in addition to the information contained in level I1. Participants assigned level I3 receive full information about the number (and value) of the 5-cent coins in the jar, in addition to the information contained in level I2. Thus I3 participants are fully informed about the number and value of 1-euro, 20-cent and 5-cent coins in the jar. No participant receives information about the number (or value) of the 1-cent coins in the jar.

To summarize, all participants have some, but incomplete information about the value of a jar from viewing and handling the jar for 15 s. Information levels I1 through I3 are cumulative, such that participants with higher information levels have all the information of participants with lower information levels, plus additional information, and are thus strictly better informed than participants with lower information levels. Designate as $V_1$, $V_{0.2}$, $V_{0.05}$ and $V_{0.01}$ the value of 1-euro, 20-cent, 5-cent and 1-cent coins in a jar. Then, depending on information level, participants have the following information about a non-stochastic lower bound of a jar’s buyback value *BBV*^4^:

- I0: lower bound equals 0
- I1: lower bound equals $V_1$
- I2: lower bound equals $V_1+V_{0.2}$
- I3: lower bound equals $V_1+V_{0.2}+V_{0.05}$.

### Procedure

The experiment consists of six sessions with 24 participants each, conducted on February 22 and 23, 2017, in the Innsbruck EconLab. The 144 participants were recruited from a standard student participant pool using hroot (Bock et al. 2014) and the experiment was conducted using GIMS 7.4.16 (Palan 2015), programmed in z-Tree 3.6.7 (Fischbacher 2007).

Half of the six sessions employ a call auction (CA), the other half a continuous double auction (CDA) trading protocol. In each session, participants arrive outside the lab and, after an experimenter has checked their IDs, are randomly assigned to workstations in the lab. An experimenter then reads out aloud the instructions on the respective trading mechanisms, with participants reading along using personal sets of paper copies of the instructions. Participants retain these paper copies for the entire experiment.^5^ Participants then complete a trial period to get acquainted with the trading interface. Following that, we hand out a second set of instructions that contains information on the jars, on the tasks to perform in the experiment, and on the payoff calculation.

The 24 participants in each session are split into three groups of eight participants each. These groups remain fixed throughout the experiment (partner matching). A session consists of 12 trading periods, structured into four blocks of three periods each (one block for each jar). At the beginning of each block, the first participant in each group receives one of the four jars, may view and handle it for 15 s and then has to hand it on to the next participant in the group, until all eight participants have had a chance to inspect the jar. Participants then submit estimates of the jar value on their respective computers. In each group, two participants each then receive information levels I0 through I3, such that each information level is represented twice in each group of eight. After having received this information, participants submit updated estimates of the jar value. They do so again at the beginning of the second and third periods in each block of three periods. The estimates are incentivized as follows: for each estimate that is within ${\pm }\,5\%$ of the true value they receive 20 cents, for each estimate that is within ${\pm }\,15\%$ they receive 10 cents, and for each estimate that is within ${\pm }\,25\%$ they receive 5 cents.

After they have submitted their estimates, participants are each endowed (virtually, on the computer) with 5 jars and an amount of experimental euros averaging twice the value of the 5 jars, while ensuring that participants cannot calculate the jar value from their cash endowment.^6^ The ratio of outstanding cash to the value of outstanding jars, which we will refer to as the cash-to-jar ratio, thus is 2.^7^ This ensures that traders are able to make transactions at reasonable frequencies and prices but it is also reasonably low to avoid biasing our results by cash endowment effects (see Kirchler et al. 2012; Noussair and Tucker 2016 and the references therein for evidence on the effect of cash endowments on mispricing). Participants then trade jars for cash for 3 min both in the CA and in the CDA treatments. Unexecuted orders can be canceled without cost at any time, and are executed according to price followed by time priority. Shorting stocks and borrowing money is not possible. No interest is paid on cash and there are no transaction costs.

Periods within a block are independent in the sense that participants’ endowments are reset to the same starting values at the beginning of every period. Procedures follow the same pattern across blocks, except that traders’ information levels and the jar they trade change (every trader receives information level I0 in one block, I1 in one block, I2 in one block and I3 in one block). Participants are fully and publicly informed about the procedures just outlined.

**Fig. 2:**
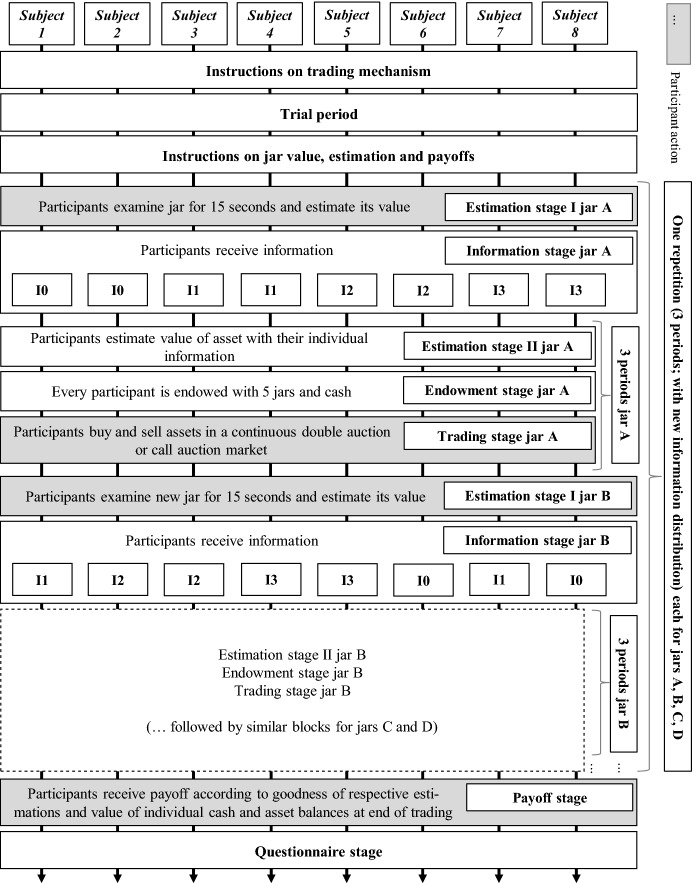
Structure of an experimental session

Finally, we ask participants to fill in a questionnaire inquiring about their gender, age, study program major, and elicit the general and the financial sub-domain risk-preference questions of Dohmen et al. (2011). The questionnaire is followed by payment. Participants’ final payoff is determined by randomly drawing one period, summing the value of final jar holdings and cash holdings, dividing by an exchange rate of 30 and adding the earnings from the estimation task. Payment is handed over individually and privately and participants are asked not to divulge details about the experiment to other students. The experiment lasted approximately 75 min and the average payment was € 16.02 per participant (s.d. 3.19). Figure 2 illustrates the session structure.

### Discussion of design choices

Before continuing to the discussion of our experimental results, we wish to take a moment to discuss some of our design choices. We accordingly structure this section by topic.

*Independence of estimates* Several authors caution that some conditions have to be met for crowd estimates to outperform other forecasting mechanisms. Surowiecki (2004) for example argues that individuals not only need to have different opinions about the issue in question, they also need to be able to make independent guesses. Similarly, Herzog and Hertwig (2011) recommend mixing participants with different backgrounds and to ask for their opinions independently. They even suggest deliberately perturbing crowd members’ original opinions by influencing them in one way or the other. Lorenz et al. (2011) note that care needs to be taken when sharing information with estimators, since “even mild social influence can undermine the wisdom of the crowd effect in simple estimation tasks” (Lorenz et al. 2011, 9020). We account for these insights by giving participants no misleading cues regarding jar values and by making them judge the jar values for themselves, privately and independently. We furthermore ‘perturb’ (in an unbiased sense) their unaided assessments by providing them with differing levels of information.

*Order of estimate elicitation* One potential concern with our design is that market prices are always obtained after the elicitation of individual estimates. These market prices thus are based on trading choices made by subjects who have previously considered the question of what they estimate the value of the asset to be. If markets simply aggregated traders’ expectations, market prices could never be worse predictors of true jar values than the aggregate of individual estimates. We agree with this line of reasoning in principle and also did not expect market prices to underperform the predictive ability of aggregated individual estimates. At the same time, this paper’s contribution lies in demonstrating whether market prices not only perform on par with individual estimates, but actually outperform them—and if so, by how much. To identify the answers to this question, we made sure subjects received no additional information between the elicitation of individual estimates and trading in the market. The trading phase takes place immediately following the post-information jar value estimate, with essentially no time in between (except for possibly a few seconds while faster participants have already submitted their estimates and are still waiting for slower participants to submit theirs). More importantly, participants receive no new information between their post-information estimate and the start of trading, such that they must base their estimate and their actions at the beginning of the trading phase on the same information.

Still, one could argue, would it not make sense to also explore the performance of market prices and individual estimates in the reverse order? In other words, should we have run additional experimental sessions where we first let experimental participants trade in markets and then elicited their individual estimates of jar values? Unfortunately, first, this is not possible in the way our experiment is designed. Note that during the trading phase, participants receive new information regarding the jar value (or at least regarding other participants’ estimates of the jar value) from observing the market prices and trading behavior. If we were to reverse the tasks and let participants trade before asking them to estimate the jar value, the estimate would invariably be influenced by what participants learned during the trading phase. The estimate would thus not be based solely on handling the jar and receiving information levels I0 through I3, but also on experience in a market. Second, reversing the order of tasks in our experiment would have defeated part of the purpose of our paper. Our paper aims to provide guidance regarding which aggregation mechanism should optimally be chosen in practice. For practical applications, both eliciting individual estimates and then aggregating them, and letting individuals trade in a market to observe market prices, are well-known (and commonly practiced) ways to approach the problem of obtaining an aggregate prediction of an uncertain or random variable. However, letting individuals outside of the lab first trade an asset in a market, only to then elicit their estimates, disregard the market data and only use the aggregate of the estimates to inform one’s opinion about the unknown quantity, seems a waste of time and resources and is, we believe, uncommon. Third, we believe that individuals who are asked to trade in a (prediction) market are likely to first consider what they believe the value of the asset to be. Explicitly asking them to provide an estimate of this value therefore is unlikely to materially bias their behavior in the market.

In light of these three points, we believe that our design is appropriate for answering our research questions and that reversing the order of the tasks would limit what we can learn from estimates while not materially improving what we can learn from market prices.

*Relation to theory* With this paper, we do not wish to challenge theoretical results regarding the aggregation of predictions, nor contribute to the theoretical literature in statistics/econometrics. Such studies usually need to assume some constraints on the prediction target (e.g., which distribution it is drawn from) or on estimator characteristics (e.g., risk-preferences—see Manski 2006; Gjerstad 2004; Wolfers and Zitzewitz 2006; Ottoviani and Sørensen 2009). We instead conduct an experimental study to see which information aggregation mechanism performs best in an empirical setting, where the distribution the underlying value is drawn from, as well as the distributions of the noise terms in individual estimates, are unknown to participants, and where participants are asymmetrically informed.

*Incentives* In addition to the forecasters differing in their information levels and presumably in how they interpret this information, incentives play a crucial role. In many contexts, incentivizing forecasters to provide their best effort in forecasting is unproblematic, since the forecast solicitors can simply pay forecasters based on the distance between their respective forecasts and the actual outcome. This is less straightforward in market experiments. While forecasters have no incentives to withhold information in the case of the individual elicitation of forecasts, do they have such incentives in prediction markets. There, their information is rendered worthless when it becomes publicly known and fully reflected in prices. When multiple trades are possible, i.e., when each trader can trade as often as s/he wants, the maximum trading profit may often be realized by revealing information (through trades) only gradually.^8^

This argument also applies in our experiment. If forecasts derived from market experiments do a good job of predicting the underlying and unknown value, this is *despite* the fact that the traders have incentives to withhold their information (particularly when it is superior) from other market participants so they alone can profit from it. In light of these considerations, we expect participants in our markets to reveal their information only gradually. This implies that price efficiency will improve over time within trading periods, such that later prices will be more informative than earlier ones.

## Results

### Individual behavior

We begin by exploring participants’ estimates. Participants provide one estimate for the value of the jar they are about to trade prior to receiving information level I0, I1, I2 or I3, and then, after they have received information, provide another estimate at the beginning of each of the three periods of trading the jar. We first look at their estimates prior to trading, i.e., in the first period they are exposed to a particular jar, after they have looked at the jar, but before starting to trade the jar (Estimation stage I in Fig. 2). These estimates are based on the ambiguous information from handling the jar, but not on information they may infer from trading with other participants.

Overall, participants underestimate the value of the coins in the jars. After having looked at and handled a jar for 15 s, but before receiving explicit information about the coins in the jar, participants underestimate the true mean jar value of € 25 by an average of € 7.09 ($t(575)=-21.425, p=0.0000$). After receiving information, this underestimation shrinks to € 3.94 ($t(575)=-15.894, p=0.0000$). Male and female participants underestimate by € 6.66 and € 7.43 (gender difference: Welch $t(568.27)=1.1728, p=0.2414$) before receiving information, respectively, and by € 3.68 and € 4.15 after (Welch $t(573.73)=0.9717, p=0.3316$).^9^

#### Result 1

*Participants underestimate jar values. There is no significant gender difference in estimate deviations*.

For our subsequent analyses, we define a participant’s jar value estimate deviation *Dev* as:

1 $$\begin{aligned} Dev^{\theta }=ln\left( \frac{Estimate^{\theta }}{BBV}\right) \end{aligned}$$

Here, $\theta \in \{pre, post\}$ signifies whether the estimate was made prior to (*pre*) or after (*post*) revelation of explicit information about the jar value (i.e., I1, I2, I3). $Dev^{\theta }$ thus measures the log percentage deviation of estimates from fundamental value.^10^

**Table 2:** OLS regressions of log jar value estimate deviation *Dev*, before (*pre*) and after (*post*) information provision

	$Dev^{pre}$	$Dev^{post}$	$Dev^{post}$
Intercept	$-\,0.277$*	$-\,0.343$***	$-\,0.362$***
	(0.161)	(0.109)	(0.059)
BBV	$-\,\,0.019$***	$-\,0.006$	$-\,0.004$*
	(0.006)	(0.004)	(0.002)
JarNo	$0.135$***	$0.058$***	$0.052$***
	(0.014)	(0.009)	(0.005)
JarPeriod			$0.023$***
			(0.007)
I1		$0.061$**	$0.036$**
		(0.029)	(0.015)
I2		$0.141$***	$0.096$***
		(0.029)	(0.015)
I3		$0.362$***	$0.293$***
		(0.029)	(0.015)
R$^2$	0.165	0.279	0.247
Adj. R$^2$	0.162	0.273	0.244
RMSE	0.372	0.248	0.227
Num. obs.	576	576	1728

***$p<0.01$; **$p<0.05$; *$p<0.1$. Standard errors in parentheses

Table 2 regresses *Dev* on jar value, on participants’ experience in judging jar value and on their information level (JarNo equals 1 for the first jar a participant sees, 2 for the second, etc.; JarPeriod equals 1 for the first period of trading a particular jar, 2 for the second, etc.).^11^ The table documents, first, that estimates of participants who have access to better information are closer to true jar values. The coefficients of the information level dummy variables are positive, statistically significant, and increase monotonously from I1 through I3. In fact, a comparison of the coefficient of I3 with that of the intercept shows that information of level I3 eliminates most of the underestimation we observe. This observation informs our first verdict regarding Hypothesis 4.^12^

#### Result Hypothesis 1(a)

The higher the information level participants have access to, the lower their estimation error.

Table 2 also shows, second, that more valuable jars’ values tend to be underestimated to a greater extent, at least before participants receive information (see the highly significant, negative coefficient of *BBV* in the first data column of Table 2).

Table 2 shows, third, that participants’ forecasts improve as they gain experience both across and within different jars. If JarNo $=$ 2, for example, this implies that this is the second jar a participant has encountered in the experiment. We find that both (i) gaining experience across different jars and (ii) observing the market across periods of trading the same jar helps participants forecast better. This allows us to come to a first verdict regarding Hypothesis 2.

#### Result Hypothesis 2(a)

Participants’ estimates improve over time, both within and across jars.

#### Estimate aggregation

We first analyze the best way to aggregate participants’ value estimates. We start by using (1) the arithmetic average, (2) the geometric average, and (3) the median values of participants’ estimates.

**Fig. 3:**
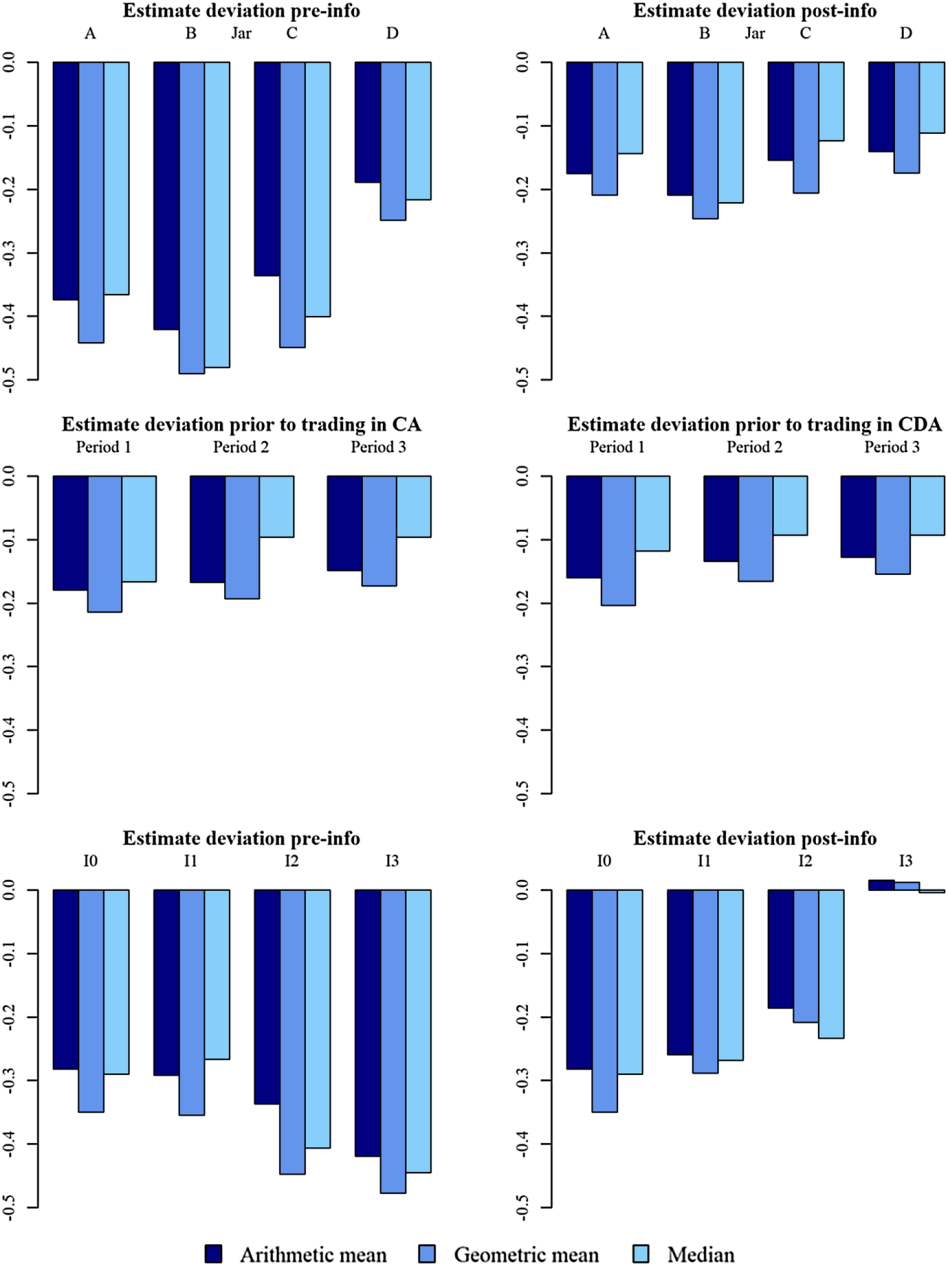
Arithmetic and geometric mean and median log estimate deviation in units of *BBV* by jar, period and information level

The three rows in Fig. 3 illustrate estimate deviations over jars, periods and information levels, respectively, using arithmetic and geometric means and the median. Overall, we find that the arithmetic mean and the median lead to very similar aggregates for participants’ estimates and that neither is clearly superior to the other. The geometric mean turns out to be farther away from *BBV* in almost all cases, but only by a very narrow margin.

The first row in Fig. 3 shows aggregated estimate deviation for each of the four jars before information is received in the left-hand panel, and after information has been received in the right-hand panel. Clearly, the information provided improves the average estimation quality, as estimation errors decrease by on average about one half. The difference is highly significant for all jars (paired *t*-tests, $t(143)\le -3.192, p<0.0017$).

The second row shows aggregated estimate deviation, pooled over all jars, for each of the three periods that participants trade the same jar. It provides (weak) evidence for some learning, as absolute estimate deviations decline slightly with experience.

The third row in Fig. 3 shows participants’ estimates depending on information level. The right-hand panel documents that higher information levels correspond to lower estimate deviation, but that only I3 participants come close to estimating jar values correctly. While I1 does not suffice to significantly improve the quality of estimates (I0 vs. I1, Welch two-sample $t(770.75)=-1.739, p=0.0824$), the information contained in I2 lowers the estimation error by about one third (I0 vs. I2, $t(638.67)=-5.066, p=0.0000$). I3 is the only information level which allows essentially accurate estimates of the coin value in the jars (I0 vs. I3, $t(460.06)=-16.967, p=0.0000$).

Interestingly, the bottom left-hand panel of Fig. 3 seems to show estimates worsening with increasing *subsequently* received information. In other words participants who, subsequent to submitting their estimates, receive information level I3 seem to have worse pre-info estimates than those subsequently receiving I0. The reason for this artifact lies in the design of our experiment: each participant receives each information level exactly for one jar (in different 3-period blocks). The order in which participants receive the information levels is randomized. However, a participant who is currently estimating the value of the jar for which she will, after the estimate, receive I0 information, may in previous blocks already have seen higher information levels (i.e., if the current jar is her fourth, she has already received I1, I2, and I3 for the three jars she traded previously). Her estimate for the fourth jar (where she will then receive information level I0) will likely be better (due to learning effects from earlier jars) than the estimate of another participant, who will (after the estimate) receive I3 information, but has never before received such high-quality information (since for his previous three jars he received only I0, I1 and I2). Especially for the second jar, the estimate of a participant who for her first jar obtained I0 may deviate strongly from the estimate of a participant who for his first jar obtained I3. The data shown in the bottom left-hand panel provides evidence supporting this explanation.

**Fig. 4:**
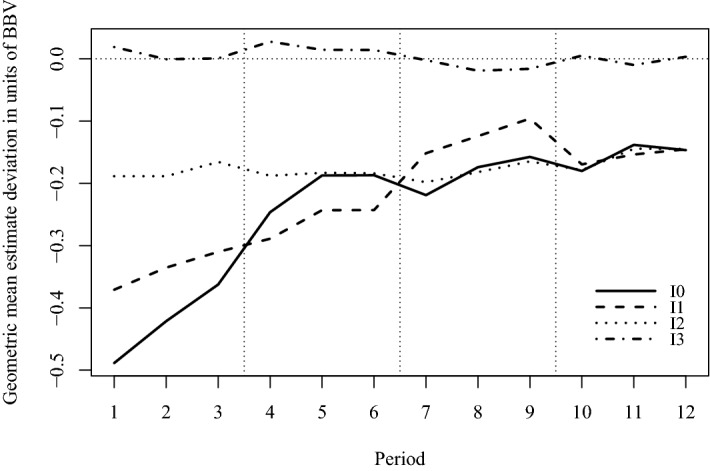
Geometric mean estimate deviation in units of *BBV* across periods, by InfoLevel. Blocks of periods with different jars are distinguished by vertical dotted lines

Figure 4 displays estimates by InfoLevel and across periods. It documents several noteworthy patterns. First, I3 participants’ estimates lie close to, and are unbiased around, the *BBV* (Wilcoxon signed rank test cannot reject median difference equal to 0, $V=42798$, $p=0.1705$). Second, all other estimate deviations are significantly different from zero (Wilcoxon signed rank tests for each information separately all yield $p=0.0000$) and from I3 (Wilcoxon signed rank tests separately comparing I3 to the other information levels all yield $p=0.0000$). Third, I2 estimates fall short of the true jar value by about 20% (5 euros), with no material learning either across periods within individual jars, or across jars. The quality of I2 estimates also seems to constitute an upper bound on the estimate precision participants with lower information levels can achieve through experience. While the estimates for levels I0 and I1 are below those of I2 until around periods 4 to 6, they are relatively similar to I2 in the final quarter of the experiment, after participants have gained sufficient experience (Wilcoxon signed rank tests separately comparing I2 to I0 and to I1 yield $p>0.2$). Thus, information dissemination seems to work to a degree that reflects the second highest information level, but not the highest. This informs our second result regarding Hypothesis 1.

##### Result Hypothesis 1(b)

Participants with the highest information level submit significantly more precise estimates than all other information levels. For lower information levels, experience can substitute information, such that experienced participants with level I0 and level I1 information submit estimates of similar precision as participants with level I2 information.

**Fig. 5:**
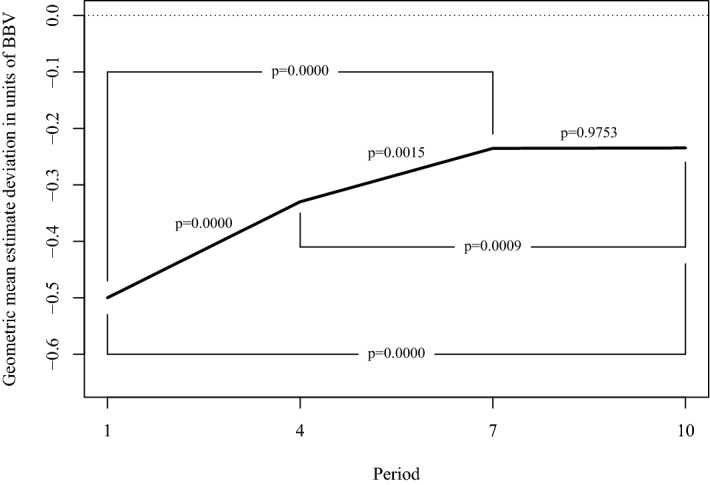
Geometric mean estimate deviation in units of *BBV* across blocks of periods and *p* values from *t* tests of equality

Figure 5 shows the average of $Dev^{pre}$, the deviation of participants’ estimates from the true jar value before receiving information about the jar value. The *p* values stem from *t* tests of the hypothesis of equal average deviations when comparing deviations at the beginning of different blocks of periods. The *p* values lacking lines to clarify which blocks are being compared compare neighboring blocks (i.e., the block starting in period 1 vs. the block starting in period 4, 4 vs. 7, and 7 vs. 10). The figure suggests that participants learn and improve their estimates between the first (period 1), second (period 4) and third (period 7) blocks, but not between the third and fourth (period 10).

A way to improve aggregate estimation quality may be to remove outliers before aggregating individual estimates. We find, however, that trimming participants’ estimates by removing a percentage of all observations from each tail of the estimate distribution has negligible effects on the quality of the mean (trimmed) estimate (see B for details).

#### Learning

We next turn to learning effects within blocks of three periods in which the same jar was traded. Specifically, we explore whether participants’ estimates improve over these three periods. We start by defining the absolute log deviation as:

2 $$\begin{aligned} AbsDev^\theta \equiv \left|Dev^\theta \right|. \end{aligned}$$

For each participant and jar, we then define $\Delta AbsDev_t$ as the change in absolute log estimate deviation from one period to the next (period 1 to period 2 and period 2 to period 3 for trading the same jar), after participants have received information, as shown in Eq. (3):

3 $$\begin{aligned} \Delta AbsDev_t\equiv AbsDev_{t+1}^{post}-AbsDev_{t}^{post}. \end{aligned}$$

We then regress $\Delta AbsDev_{t\in {1,2}}$ on participants’ absolute log estimate deviation after they receive information in the first period of trading a new jar ($AbsDev_{t=1}^{post}$), interacted with dummy variables for whether the learning took place over the first or over the second period. The coefficients of these regressors can thus be interpreted as the fraction of the initial absolute log estimate deviation that participants correct due to learning from trading. We report the results in the first content column of Table 3.

**Table 3:** OLS regressions of $\Delta AbsDev_t$ on initial absolute log estimate deviation after information revelation, interacted with period dummy variables (but no intercept) and other regressors

	$\Delta AbsDev_t$	$\Delta AbsDev_t$	$\Delta AbsDev_t$
$AbsDev^{post}_1\times P1$	$-\,0.218$***	$-\,0.225$***	$-\,0.260$***
	(0.013)	(0.015)	(0.024)
$AbsDev^{post}_1\times P2$	$-\,0.068$***	$-\,0.075$***	$-\,0.111$***
	(0.013)	(0.015)	(0.024)
CA		0.009	0.004
		(0.006)	(0.007)
Ability		0.002	$-\,0.003$
		(0.002)	(0.004)
Jar A			0.012
			(0.011)
Jar B			0.009
			(0.012)
Jar C			0.013
			(0.012)
Jar D			0.009
			(0.011)
Female			$0.013$*
			(0.007)
$\hbox {R}^2$	0.205	0.209	0.214
Adj. $\hbox {R}^2$	0.204	0.206	0.208
RMSE	0.114	0.114	0.113
Num. obs.	1152	1152	1152

***$p<0.01$; **$p<0.05$; *$p<0.1$. Standard errors in parentheses

The coefficients document that the estimate after the first trading period is about 22 percentage points closer to *BBV* than the estimate before the first trading period, and that the second trading period yields another improvement of about 7 percentage points. In the second column we add a dummy variable for the call auction sessions and a measure of participants’ estimation ability. We define the latter as:

4 $$\begin{aligned} Ability \equiv ln\left( \frac{AbsDev_{t=1}^{post}}{avg\left( AbsDev_{t=1}^{post}\right) }\right) , \end{aligned}$$

where ${AbsDev}_{t=1}^{post}$ is the participant’s absolute log estimate deviation for a particular jar, after receiving information and before trading in the first period of trading this jar, and $avg\left( {AbsDev}_{t=1}^{post}\right)$ is the average of the same variable over all participants. *Ability* thus is the (log) percentage outperformance of the participant’s estimate relative to the average estimate by all participants. Adding these two variables to our regression model does not affect the discovered learning effects. In the third column, we also add dummy variables for the four different jars, as well as gender, which shows that learning does not differ much (by approximately 1%) between female and male participants. Overall, none of the more complex specifications materially improves the explanatory power (i.e., $R^2$) of the first regression model. This informs our second finding regarding Hypothesis 2.

##### Result Hypothesis 2(b)

Participants’ estimates of jar value improve by about 22 percentage points over the first period of trading, and by another about 7 percentage points over the second. Neither the trading mechanism nor participants’ estimation ability or gender materially moderates this learning process.

### Market-level results

**Fig. 6:**
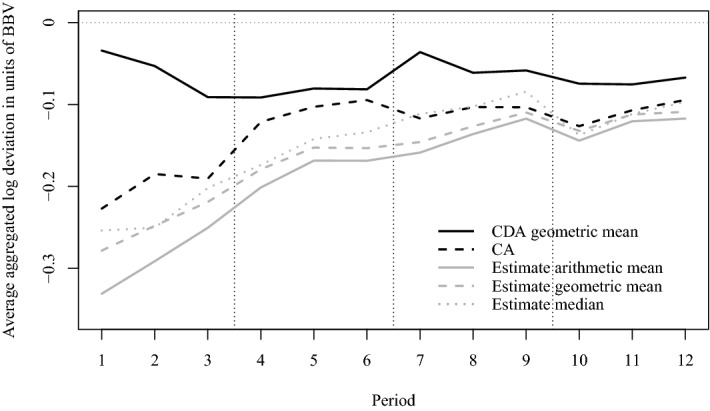
Average of period arithmetic and geometric mean and median log estimate deviation as well as of geometric mean CDA transaction prices and CA prices from *BBV* (in units of *BBV*) over trading periods

We now turn to the comparison of the two market mechanisms Call Auction vs. Continuous Double Auction. Figure 6 plots the average log deviation of transaction prices over time, measured in periods. For comparison purposes, we also plot the average deviations of mean and median estimates (post information revelation). If participants learned across jars and over time, we would expect a monotonous upward trend. There is no significant evidence for such learning for arithmetic and geometric mean estimates (Mean-Kendall trend test, using the average estimate within each period and session as one observation, both $z(216)=-1.5247, p=0.1273$), median estimates ($z(216)=-1.944, p=0.0519$), CDA prices ($z(108)=1.383, p=0.1666$) or CA prices ($z(108)=-0.927, p=0.3537$).

**Fig. 7:**
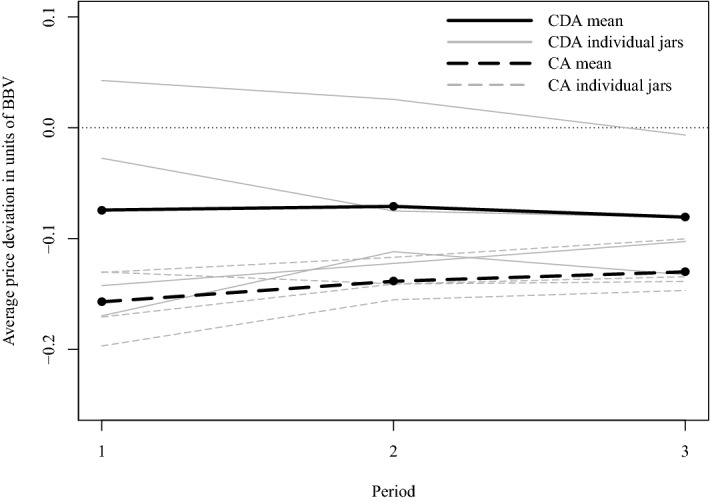
Period averages of geometric mean log continuous double auction (CDA) price deviation and of log call auction (CA) price deviation, in units of *BBV*

Figure 7 plots individual jars’ and the mean’s price development over the three periods each jar is traded for, separately for CA and CDA. In neither treatment do we observe learning across periods within a jar, but we see that prices deviate less in the CDA treatment than in the CA treatment.

**Fig. 8:**
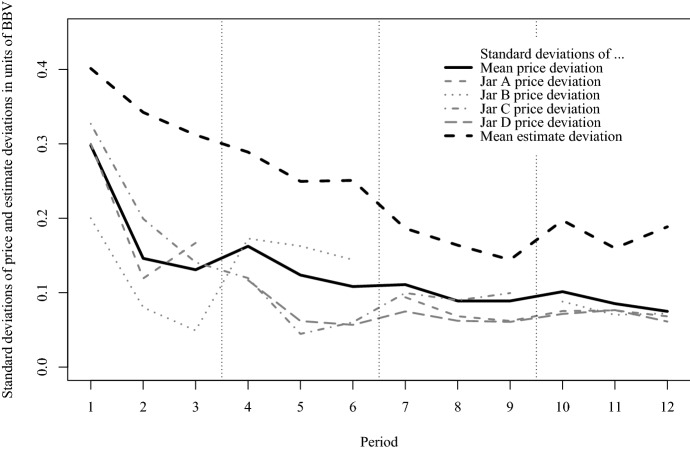
Average standard deviation of the log deviations of transaction prices (and individual estimates after information revelation) from *BBV* (in units of *BBV*) over trading periods

Figure 8 plots the standard deviation of transaction price deviations from *BBV* over the trading periods for each jar. It shows a downward trend, indicating harmonization of participants’ estimates in light of their observations in the market. It also contains a line showing estimate deviations, which follow a similar pattern, yet remain at a higher level. This documents that market prices offer less noisy predictions of jar value than individual estimates.

### Participant earnings

Table 4 reports a regression analyzing the percentage change in participants’ wealth, $\Delta Wealth$:

5 $$\begin{aligned} \Delta Wealth=\left( \frac{FinalCash+FinalJars\cdot BBV}{InitialCash+InitialJars\cdot BBV}-1\right) \cdot 100. \end{aligned}$$

We multiply by 100 to scale up the regression coefficients for better legibility. Regarding the regressors, we again use $AbsDev^{post}$, the log deviation from *BBV* of participants’ estimates of the jar value after information provision in period 1 and at the beginning of the period in periods 2 and 3 of trading each jar. This variable constitutes an inverse measure of participants’ precision in estimating jar values, incorporating the information provided by the experimenter and the information gathered by observing (and participating in) trading. $AbsDev^{pre}$, a similar measure as $AbsDev^{post}$, is calculated only once for each jar (when participants first estimate the jar value) and is kept constant within the three periods of trading of each jar. It thus measures a participant’s estimation ability, bar explicit information about the jar value (from information levels I1 through I3) and bar learning effects from trading. *Female* is a dummy variable for participant gender (using the obvious coding), and I1 through I3 are dummy variables denoting a participant’s information level in any given period.

**Table 4:** Regressions of percentage change in subjects’ wealth (evaluated at *BBV*) over the course of a period

	$\Delta Wealth$	$\Delta Wealth$	$\Delta Wealth$
*Intercept*	$2.295$***	$1.541$***	$1.444$***
	(0.290)	(0.317)	(0.320)
$AbsDev^{post}$	$-\,6.837$***	$-\,5.140$***	$-\,6.395$***
	(0.766)	(0.810)	(0.894)
$AbsDev^{pre}$			$1.313$***
			(0.460)
*Female*	$-\,1.449$***	$-\,1.547$***	$-\,1.574$***
	(0.352)	(0.353)	(0.354)
*I*1		$-\,0.177$	$-\,0.250$
		(0.270)	(0.272)
*I*2		$-\,0.042$	$-\,0.319$
		(0.259)	(0.276)
*I*3		$1.981$***	$1.481$***
		(0.384)	(0.396)
R$^2$	0.171	0.204	0.211
Adj. R$^2$	0.170	0.202	0.208
RMSE	4.038	3.961	3.945
Num. obs.	1728	1728	1728

***$p<0.01$; **$p<0.05$; *$p<0.1$. Standard errors adjusted for 144 clusters at the subject level in parentheses

The highly significant coefficients for $AbsDev^{post}$ in Table 4 show that participants with greater $AbsDev^{post}$, and thus a relatively low-quality estimate of *BBV*, end up with lower wealth than participants who are more successful in estimating jar value. This is in line with our Hypothesis 3.

#### Result Hypothesis 3

The greater the absolute deviation of traders’ informed estimates from true jar values, the lower is traders’ final wealth.

When we add information level dummy variables, it is interesting to see that the coefficient for I3 is significant even after controlling for $AbsDev^{post}$. This is caused by the non-linearity of the relationship between information level and final profits. I3 participants earn 3.29% more than I0-I2 participants on average (see Figure OA.1 in the online appendix), while the lower information levels I1 and I2 do not confer significantly higher final wealth positions on their recipients. We conclude that our Hypothesis 4 finds partial support.

#### Result Hypothesis 4

Traders with access to the highest information level earn excess profits. Traders with lower information levels do not significantly outperform uninformed traders in terms of final wealth.

The lower earnings of female participants may stem from the fact that female participants end each period holding on average 4.47 jars, while male participants average 5.66. Remember that jars are on average undervalued. Male traders thus tend to be net buyers of jars, paying less than *BBV*, but earning *BBV* for each jar bought in this way.^13^ The rightmost column finally adds $AbsDev^{pre}$ to explore whether participants’ innate estimation ability (“pure” ability, without information or prior experience with the jar being traded) helps them outperform. This seems not to be the case. The effect of the positive coefficient of $AbsDev^{pre}$ is in fact nearly entirely compensated by the larger (in absolute terms) negative coefficient of $AbsDev^{post}$ in this model.

Table 5 shows the average log deviations from *BBV* when using different mechanisms to aggregate predictions of the true jar value. Period refers to the period within a block of trading a single jar and the table lists averages across all jars. The aggregation methods summarized in the table are the midpoint of the bid-ask spread at the end of the trading period in the CDA (CDA mid), the closing, arithmetic and geometric mean, and median prices in the CDA (CDA close, CDA amean, CDA gmean, CDA med), the median and the arithmetic and geometric mean jar value estimates after receiving information (Est. med, Est. amean, Est. gmean), and the price in the CA (CA). In this and the following paragraphs, we focus on the first period, as in many situations outside of the lab where good estimates of an unknown quantity are required, it is impractical to let participants trade/estimate for multiple periods. The table shows that the absolute deviation is lowest when using the midpoint of the bid-ask spread in the continuous double auction.

**Table 5:** Log deviation from BBV (in %) resulting from different aggregation mechanisms. Columns are sorted in ascending order by absolute deviation in the first period of each block

Period	CDA mid	CDA close	CDA gmean	CDA med	Est. med	CA	Est. gmean	Est. amean
1	− 4.314	− 5.092	− 5.900	− 6.067	− 16.114	− 16.651	− 18.739	− 20.875
2	− 6.250	− 8.813	− 6.753	− 6.823	− 13.909	− 13.671	− 16.298	− 17.907
3	− 4.031	− 8.245	− 7.447	− 7.622	− 11.888	− 13.216	− 15.003	− 16.338
All	− 5.177	− 7.384	− 6.700	− 6.837	− 13.970	− 14.513	− 16.680	− 18.373

‘amean’ stands for the arithmetic mean, ‘close’ for the market closing price, ‘gmean’ for the geometric mean, ‘med’ for the median and ‘mid’ for the bid-ask spread midpoint at market close

**Table 6:** *p* values from pairwise t-tests comparing the log deviations from BBV resulting from different aggregation mechanisms using only data from the first period within a block

	CDA mid	CDA close	CDA gmean	CDA med	Est. med	CA	Est. gmean	Est. amean
CDA mid		0.9777	0.8417	0.8734	0.0013	0.0008	0.0000	0.0000
CDA close	0.9777		0.8420	0.8255	0.0001	0.0000	0.0000	0.0000
CDA gmean	0.8417	0.8420		0.7131	0.0013	0.0008	0.0000	0.0000
CDA med	0.8734	0.8255	0.7131		0.0005	0.0003	0.0000	0.0000
Est. med	0.0013	0.0001	0.0013	0.0005		0.8445	0.2343	0.0453
CA	0.0008	0.0000	0.0008	0.0003	0.8445		0.3401	0.0704
Est. gmean	0.0000	0.0000	0.0000	0.0000	0.2343	0.3401		0.1807
Est. amean	0.0000	0.0000	0.0000	0.0000	0.0453	0.0704	0.1807	

‘amean’ stands for the arithmetic mean, ‘close’ for the market closing price, ‘gmean’ for the geometric mean, ‘med’ for the median and ‘mid’ for the bid-ask spread midpoint at market close

Table 6 displays *p* values when comparing the average deviations resulting from the use of the aggregation mechanisms listed in Table 5. Table 6 only uses data from the first period within a block. Furthermore, the rows and columns in the table are sorted by increasing absolute deviation in the first period.^14^^,^^15^ The data documents that predictions based on CDA data clearly outperform the CA and mean and median estimates. The differences within the CDA are not significant. When relying only on estimates, the median weakly outperforms the arithmetic, but not the geometric mean as an aggregation mechanism. We thus see Hypothesis 5 largely supported.

#### Result Hypothesis 5

CDA prices are closest to true jar values. CA prices and individual estimates perform significantly worse. Limiting the analysis to the simple estimates, aggregation using the median performs best.

Note that one caveat to our finding of outperformance of the CDA is that prices and estimates in all of our sessions tend to be below *BBV*. This implies that the best estimates of true jar values are, at the same time, also the highest estimates. Given our experimental data, we thus cannot rule out that the CDA generally produces higher, instead of better, predictions than other aggregation methods. Testing whether the CDA also outperforms in cases where participants generally overestimate the target value will require additional data.^16^

## Conclusion

The present paper reports on a lab experiment studying different mechanisms for aggregating dispersed information. We use the controlled conditions of the experimental laboratory to compare the quality of predictions of an unknown quantity stemming from (1) participants’ estimates, (2) continuous double auction, or CDA, market prices and (3) call auction, or CA, market prices. We find that prices in a CDA constitute the best aggregation mechanism, characterized by the lowest prediction error.

The outperformance of the CDA is in line with the recent successes of prediction markets and it supports the use of market mechanisms for information aggregation. However, while the CDA outperforms the other aggregation mechanisms, it is at the same time the most complex of the mechanisms employed in our study. A simple estimate (even with incentivization) can be elicited very quickly and using any medium (verbal, paper, online). Conducting a continuous auction market requires considerable investment both in terms of the solicitor’s infrastructure and of participants’ time. Furthermore, the possibility of observing no or only few trades—and the potential cost of guarding against this eventuality—should also be taken into consideration. Whether these additional monetary and non-monetary costs are justified cannot be answered in general. Instead, this question needs to be answered on a case-by-case basis, weighing the CDA’s greater costs against the benefits that can be derived from the greater forecast precision it offers.

We hope that, in addition to our results per se, our methodology may also help future researchers. Having participants handle and estimate the value of multiple types of coins in a jar and providing them with varying levels of information about the coins in the jar allows for studying both ambiguity and risk, and for implementing a number of valuable treatment variations. For future research, it would for example be interesting to apply the approach of Budescu and Chen (2014) to our setting. They compare individual participants’ performance with that of the group and then let only above-average participants (i.e., ‘experts’) interact with each other in a second round. As pointed out in the previous section, we would also consider it a worthwhile endeavor to test the performance of our aggregation mechanisms using assets which experimental participants tend to over-instead of underestimate.

## Electronic supplementary material

Below is the link to the electronic supplementary material.

Supplementary material 1 (pdf 399 KB)

## Footnotes

1
Mannes et al. (2012) use Jensen’s (1906) inequality to prove that the absolute forecast error of the average estimate must be smaller than or equal to the average of the individual estimates’ absolute forecast errors. For the task of arriving at a point forecast, this implies that the average over a set of estimates is a (probabilistically) better—i.e., more precise—predictor of the value to be estimated than a randomly chosen element of the set of estimates.

2 Such a derivative asset may, for example, at a pre-defined maturity date, pay a fixed amount of money conditional on an underlying event having occurred (e.g., a contract that pays $ 1 if politician X gets elected). Alternatively, the asset may pay an amount that is a linear function of the underlying quantity to be estimated (e.g., a contract that pays $ *x*, where *x* is the vote share of politician X, in percent).

3 We will hereafter use expressions like “value of the coins in the jar” interchangeably with “value of the jar” or *BBV* (buyback value).

4 Of course, since participants can view and handle jars, they can instantly establish a lower bound above 0 even in I0.

5 A translation of all instructions, which were originally in German, is included in section F of the online appendix.

6 For the determination of these euro amounts, we started from two principles. First, there should be no direct correspondence between euro amount and jar value to prevent traders from inferring the latter from the former. Second, the cash-to-jar value ratio should be constant at a value of 2 across all markets. We thus obtained the euro values as follows: We randomly drew (and redrew), for each participant, cash endowments from a uniform distribution over [200, 300] experimental euros. We repeated the drawing until the ratio of total cash endowment in the market to total jar endowment value equalled, to two decimal places, 2. We thus obtain individual cash endowments which vary substantially around twice the value of the jar endowment, while ensuring that the cash-to-jar value ratio always equals 2 at the market level. See Table OA.1 in the online appendix for details. Participants are symmetrically informed that each participant is endowed with 5 jars and they are told that each participant is endowed with a euro amount that varies across participants and periods. They are not informed about details of the cash endowment determination algorithm.

7 In the related literature, this ratio is frequently also referred to as the cash-to-asset ratio.

8 See for example the model of Kyle (1985) or the work of Chamley and Gale (1994).

9 We did not expect to find a gender effect in our data ex ante, but controlled for participant gender among other participant characteristics elicited via a post-experiment questionnaire, since gender (or beliefs about it) has been shown to affect behavior in asset markets in the past (see e.g., Eckel and Füllbrunn 2015, 2017; Holt et al. 2017). We find that gender appears to play a role in several of our analyses. In the interest of transparency, we thus report on gender differences throughout this section.

10 Due to the log specification, this measure is independent of the choice of numeraire (i.e., whether one expresses prices as talers/jar or jars/taler). See Powell (2016) for details.

11 Table OA.2 in the online appendix repeats this analysis but includes participant dummy variables (albeit, to conserve space, not in the output) to give a better indication of the explanatory power of the models ($R^2>0.5$ throughout) when accounting for participant heterogeneity.

12 Further insights into this hypothesis follow later.

13 Including a measure of participants’ risk-preferences (following Dohmen et al. 2011) does not materially affect these findings.

14 We discard 7 out of our 1746 (0.4%) offers outstanding at the end of a period because they have prices of 1000 or above, which are likely not meant to be serious and even if so, would bias our results without adding valuable insights. Furthermore, we are most interested in Period 1 data and these outliers only occur in Period 3 data.

15 We end up with 4 out of 108 (3.7%) periods where we cannot calculate a bid-ask midpoint due to missing best bid or best ask values.

16 We thank an anonymous referee for pointing this out.

## References

[CR1] Berg J, Forsythe R, Nelson F, Rietz T, Plott CR, Smith VL (2008). Results from a dozen years of election futures markets research, Ch. 80. Handbook of Experimental economics results.

[CR2] Berg JE, Nelson FD, Rietz TA (2008). Prediction market accuracy in the long run. International Journal of Forecasting.

[CR3] Berg JE, Rietz TA (2003). Prediction markets as decision support systems. Information Systems Frontiers.

[CR4] Bock O, Baetge I, Nicklisch A (2014). hroot: Hamburg registration and organization online tool. European Economic Review.

[CR5] Brown WO, Sauer RD (1993). Fundamentals or noise? Evidence from the professional basketball betting market. The Journal of Finance.

[CR6] Bruce RS (1935). Group judgments in the fields of lifted weights and visual discrimination. The Journal of Psychology.

[CR7] Budescu DV, Chen E (2014). Identifying expertise to extract the wisdom of crowds. Management Science.

[CR8] Budescu DV, Yu H-T (2006). To Bayes or not to Bayes? A comparison of two classes of models of information aggregation. Decision Analysis.

[CR9] Chamley C, Gale D (1994). Information revelation and strategic delay in a model of investment. Econometrica.

[CR10] Choo L, Kaplan TR, Zultan R (2019). Information aggregation in Arrow–Debreu markets: An experiment. Experimental Economics.

[CR11] Clemen RT (1989). Combining forecasts: A review and annotated bibliography. International Journal of Forecasting.

[CR12] Davis-Stober CP, Budescu DV, Dana J, Broomell SB (2014). When is a crowd wise?. Decision.

[CR13] Diamond DW, Verrecchia RE (1981). Information aggregation in a noisy rational expectations economy. Journal of Financial Economics.

[CR14] Dohmen TJ, Falk A, Huffman D, Schupp J, Sunde U, Wagner G (2011). Individual risk attitudes: Measurement, determinants, and behavioral consequences. Journal of the European Economic Association.

[CR15] Eckel CC, Füllbrunn S (2015). Thar she blows? Gender, competition, and bubbles in experimental asset markets. The American Economic Review.

[CR16] Eckel CC, Füllbrunn SC (2017). Hidden vs. known gender effects in experimental asset markets. Economics Letters.

[CR17] Fischbacher U (2007). z-Tree: Zurich toolbox for ready-made economic experiments. Experimental Economics.

[CR18] Forsythe R, Lundholm R (1990). Information aggregation in an experimental market. Econometrica.

[CR19] Gjerstad, S. (2004). *Risk aversion, beliefs, and prediction market equilibrium*, University of Arizona, Department of Economics Working Paper 04-17.

[CR20] Gordon KH (1924). Group judgments in the field of lifted weights. Journal of Experimental Psychology.

[CR21] Gruca T, Berg JE, Cipriano M (2003). The effect of electronic markets on forecasts of new product success. Information Systems Frontiers.

[CR22] Herzog S, Hertwig R (2011). The wisdom of ignorant crowds: Predicting sport outcomes by mere recognition. Judgment and Decision Making.

[CR23] Holt CA, Porzio M, Song MY (2017). Price bubbles, gender, and expectations in experimental asset markets. European Economic Review.

[CR24] Huber J (2007). ‘j’-shaped returns to timing advantage in access to information—experimental evidence and a tentative explanation. Journal of Economic Dynamics & Control.

[CR25] Huber J, Kirchler M, Sutter M (2008). Is more information always better? Experimental financial markets with cumulative information. Journal of Economic Behavior & Organization.

[CR26] Jensen JLWV (1906). Sur les fonctions convexes et les inégalités entre les valeurs moyennes. Acta Mathematica.

[CR27] Kirchler M, Huber J, Stöckl T (2012). Thar she bursts—reducing confusion reduces bubbles. The American Economic Review.

[CR28] Kyle RA (1985). Continuous auctions and insider trading. Econometrica.

[CR30] Lichtendahl K, Grushka-Cockayne Y, Pfeifer P (2013). The wisdom of competitive crowds. Operations Research.

[CR31] Lorenz J, Rauhut H, Schweitzer F, Helbing D (2011). How social influence can undermine the wisdom of crowd effect. Proceedings of the National Academy of Sciences.

[CR32] Lorge I, Fox DDJBM (1958). A survey of studies contrasting the quality of group performance and individual performance. Psychological Bulletin.

[CR33] Malone, T., Laubacher, R., & Dellarocas, C. (2009). *Harnessing crowds: Mapping the genome of collective intelligence*. SSRN working paper 1381502.

[CR29] Mannes AE, Larrick RP, Soll JB, Krueger JI (2012). The social psychology of the wisdom of crowds. Frontiers of social psychology. Social judgment and decision making.

[CR34] Manski, C. F. (2006). Interpreting the predictions of prediction markets. *Economics Letters 91*(3), 425–429, revision of NBER Working Paper 10359, March 2004.

[CR35] Noussair CN, Tucker S (2016). Cash inflows and bubbles in asset markets with constant fundamental values. Economic Inquiry.

[CR36] Ostrovsky M (2012). Information aggregation in dynamic markets with strategic traders. Econometrica.

[CR37] Ottoviani, M., & Sørensen, P. N. (2009). *Aggregation of information and beliefs: Asset pricing lessons from prediction markets*. SSRN discussion paper 1447369.

[CR38] Palan S (2015). GIMS—Software for asset market experiments. Journal of Behavioral and Experimental Finance.

[CR39] Plott CR, Sunder S (1988). Rational expectations and the aggregation of diverse information in laboratory security markets. Econometrica.

[CR40] Polgreen PM, Nelson FD, Neumann GR (2007). Use of prediction markets to forecast infectious disease activity. Clinical Infectious Diseases.

[CR41] Powell O (2016). Numeraire independence and the measurement of mispricing in experimental asset markets. Journal of Behavioral and Experimental Finance.

[CR42] Sauer RD (1998). The economics of wagering markets. Journal of Economic Literature.

[CR43] Schredelseker K (1984). Anlagestrategie und informationsnutzen am aktienmarkt. Zeitschrift für Betriebswirtschaftliche Forschung.

[CR44] Soll J, Richard B, Larrick P (2009). Strategies for revising judgment: How (and how well) people use others’ opinions. Journal of Experimental Psychology: Learning, Memory and Cognition.

[CR45] Sunder S (1992). Market for information: Experimental evidence. Econometrica.

[CR46] Surowiecki J (2004). The wisdom of crowds.

[CR47] Wolfers, J., & Zitzewitz, E. (2006). *Interpreting prediction market prices as probabilities*. SSRN Discussion paper 898597.

