# Supplementary material for: Aggregation mechanisms for crowd predictions
Source: Exp Econ. 2019 Nov 9;23(3):788–814. doi: 10.1007/s10683-019-09631-0 (PMC7591134; doi:10.1007/s10683-019-09631-0)
Supplement: Supplementary file 1 — Supplementary material 1 (pdf 399 KB) [file 10683_2019_9631_MOESM1_ESM.pdf]

Online Appendix to
"Aggregation mechanisms for crowd predictions"

Stefan Palan, Jürgen Huber, Larissa Senninger

A. Additional figures and tables

Figure OA.1 plots percentage change in participant wealth over the four information levels. The advantage of obtaining information level I3 is evident. None of the pairwise differences between $\Delta Wealth$ among I0, I1 and I2 are significant (Wilcoxon rank sum test, $p > 0.6$ for all comparisons), while all differences are significant when comparing to I3 ($p = 0.0000$ for all comparisons).

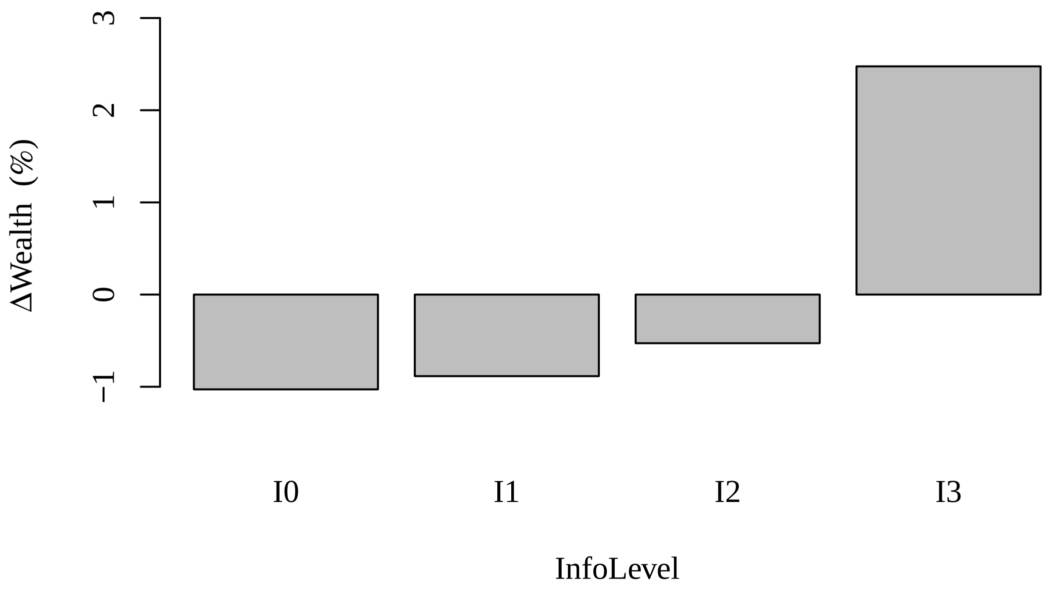

Figure OA.1: Percentage change in wealth per participant, over information levels; means over all sessions.

Table OA.1 lists the cash endowments participants received at the beginning of each period of trading a particular jar.

Table OA.1: Cash endowments

jar	A	B	C	D	Average
Participant 1	221	316	259	204	250
Participant 2	238	306	215	241	250
Participant 3	232	270	276	222	250
Participant 4	282	287	237	194	250
Participant 5	261	300	221	218	250
Participant 6	213	307	232	248	250
Participant 7	231	267	250	252	250
Participant 8	282	288	220	210	250
Cash-Jar-Ratio	1.99918	2.00017	1.99958	2.00112	

Table OA.2 repeats the analysis of Table 2, yet includes participant dummy variables (albeit, to conserve space, not in the output) to give a better indication of the explanatory power of the models ($R^2 > 0.5$ throughout) when accounting for participant heterogeneity.

	Dev^{pre}	Dev^{post}	Dev^{post}	Dev^{post}
Intercept	-0.579^{***}	-0.353^{**}	-0.585^{***}	-0.526^{***}
	(0.207)	(0.144)	(0.140)	(0.070)
BBV	-0.019^{***}	-0.010^{***}	-0.006^{*}	-0.004^{**}
	(0.005)	(0.004)	(0.003)	(0.002)
JarNo	0.135^{***}		0.058^{***}	0.052^{***}
	(0.012)		(0.008)	(0.004)
JarPeriod				0.023^{***}
				(0.005)
I1		0.061^{**}	0.061^{**}	0.036^{***}
		(0.026)	(0.025)	(0.012)
I2		0.141^{***}	0.141^{***}	0.096^{***}
		(0.026)	(0.025)	(0.012)
I3		0.362^{***}	0.362^{***}	0.293^{***}
		(0.026)	(0.025)	(0.012)
R^2	0.551	0.561	0.610	0.561
Adj. R^2	0.400	0.410	0.475	0.519
RMSE	0.314	0.223	0.211	0.181
Num. obs.	576	576	576	1728

$^{***}p < 0.01$; $^{**}p < 0.05$; $^{*}p < 0.1$. Standard errors in parentheses.

Table OA.2: OLS regressions of log jar value estimate deviation *Dev*, before (*pre*) and after (*post*) information provision. Dummy variables for individual subjects were included in the estimations but omitted in the output.

B. Analysis of trimmed mean estimates

For Figure OA.2, we calculate mean estimate deviations after removing outliers from the data. Specifically, we trim participants' estimates by removing a percentage of all observations from each tail of the estimate distribution. We then calculate the arithmetic mean estimate deviation for the trimmed data and plot it over different trim levels.[1] The shading in the background indicates ± 1 standard deviation around the trimmed mean estimates. The figure suggests that, for our data, the effect of removing outlying observations before averaging has negligible effects on the mean estimate, particularly in light of the wide standard deviation bands.

[1]Note that, at the extremes, a two-tailed trim percentage of zero implies no removal of outliers, while a percentage of 50 implies using the median estimate only.

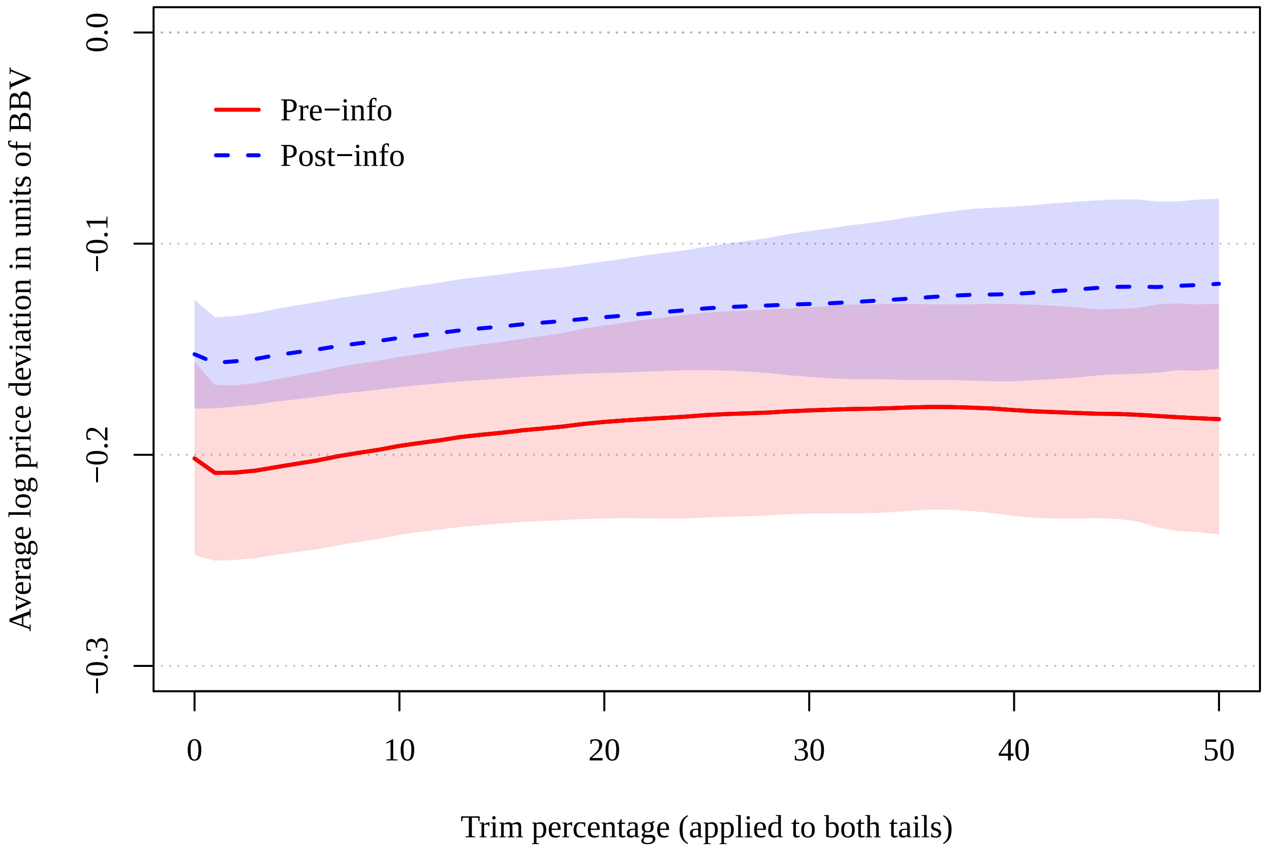

Figure OA.2: Log deviation of trimmed mean estimates from BBV (in units of BBV) over different percentages of trimming. Trimming entails removing a set percentage of all observations from each tail of the distribution prior to calculating the arithmetic mean of the remaining observations. At the two extremes, a percentage of zero implies no removal of outliers, while a percentage of 50 implies using the median estimate only. The shaded areas depict ±1 standard deviation (calculated over the four jar means).

C. Analysis of estimates before information revelation

Section 3.1.1 mentions (and Figure 3 shows) that participants' jar value estimates before receiving high-quality information (e.g., I3) are worse than their estimates before receiving low-quality information (e.g., I0). As Table OA.3 shows, this is due to participants learning from the information received in previous blocks of trading different jars. The Table uses data from periods 4, 7 and 10 (i.e., the first periods of trading each jar after the first) and analyzes as dependent variable participants' absolute log estimate deviation before receiving information. This variable, $AbsDev_t^{pre}$, is regressed on (1) dummy variables for the four jars,

(2) dummy variables for whether the participant's history contains periods with information levels I1, I2 or I3, (3) a dummy variable for whether the observations stem from a call auction market, and (4) a dummy variable for whether the participant in question was female. Note that the regressions in Table OA.3 forego the use of an intercept in favor of using all four jar dummy variables.

	$AbsDev_t^{pre}$	$AbsDev_t^{pre}$	$AbsDev_t^{pre}$	$AbsDev_t^{pre}$
Jar A	0.852^{***}	0.753^{***}	0.745^{***}	0.721^{***}
	(0.041)	(0.046)	(0.047)	(0.048)
Jar B	0.861^{***}	0.763^{***}	0.755^{***}	0.731^{***}
	(0.038)	(0.043)	(0.045)	(0.046)
Jar C	0.806^{***}	0.707^{***}	0.699^{***}	0.675^{***}
	(0.035)	(0.041)	(0.043)	(0.044)
Jar D	0.749^{***}	0.650^{***}	0.642^{***}	0.618^{***}
	(0.045)	(0.048)	(0.050)	(0.051)
JarNo	-0.145^{***}	-0.046^{*}	-0.046^{*}	-0.053^{**}
	(0.012)	(0.026)	(0.026)	(0.026)
$I1 \in$ history		-0.031	-0.031	-0.024
		(0.037)	(0.037)	(0.037)
$I2 \in$ history		-0.086^{**}	-0.086^{**}	-0.077^{*}
		(0.040)	(0.040)	(0.040)
$I3 \in$ history		-0.278^{***}	-0.278^{***}	-0.269^{***}
		(0.037)	(0.037)	(0.037)
CA			0.016	0.009
			(0.024)	(0.024)
Female				0.061^{**}
				(0.025)
R^2	0.716	0.745	0.746	0.748
Adj. R^2	0.714	0.742	0.741	0.744
RMSE	0.307	0.292	0.292	0.291
Num. obs.	576	576	576	576

$^{***}p < 0.01$, $^{**}p < 0.05$, $^{*}p < 0.1$. Standard errors in parentheses.

Table OA.3: OLS regressions of $|Dev_t^{pre}|$ on dummy variables for the jars, for having previously received information levels I1 through I3, for the trading mechanism and subject gender.

The first content column in Table OA.3 documents that participants learn across jars. The more jars they have previously traded, the lower their absolute log estimate deviation. Content column 2 refines this finding by showing that much of the learning stems from having previously received high-quality information about a jar's value. Participants who have, for previous jars, received I3 information, submit jar value estimates which are about 30% more accurate than participants who have yet to experience I3 information. Content

column 3 shows that the trading mechanism does not affect the dependent variable, while the final column suggests that male participants' estimates are about 6% more accurate than female participants' after controlling for all other variables. The R^2 values indicate an excellent model fit.

D. Analysis of the effect of estimation ability on market prices

Table OA.4 regresses the absolute log deviation of period mean and median price, closing price and closing bid-ask spread in the CDA as well as the absolute log deviation of the CA price on the period average absolute log deviation of participants' estimates before information revelation, controlling for individual jar effects. The regressions use only the first period of trading for each jar in order to isolate, as far as possible, the pure ability effect from learning across periods. The table shows that estimate quality, i.e., participants' average ability in forming estimates of jar value, improves market prices in the CA, but not in the CDA. Table OA.5 repeates the analysis with the even more stringent specification that it uses only the very first period in a session. It confirms the findings from Table OA.4.

	CDA mean	CDA median	CDA close	CDA mid	CA
Intercept	0.077	0.097*	0.110**	0.135*	0.029
	(0.061)	(0.049)	(0.046)	(0.066)	(0.036)
$AbsDev^{pre}$	0.139	0.117	0.014	−0.016	0.342***
	(0.104)	(0.084)	(0.079)	(0.113)	(0.056)
Jar 2	−0.002	−0.006	0.011	0.054	0.014
	(0.061)	(0.049)	(0.046)	(0.066)	(0.036)
Jar 3	−0.016	−0.049	−0.029	−0.050	−0.048
	(0.060)	(0.049)	(0.046)	(0.065)	(0.036)
Jar 4	−0.041	−0.050	−0.065	−0.067	0.008
	(0.064)	(0.051)	(0.048)	(0.069)	(0.038)
R^2	0.106	0.150	0.105	0.115	0.585
Adj. R^2	−0.009	0.040	−0.010	0.001	0.531
RMSE	0.128	0.103	0.097	0.139	0.077
Num. obs.	36	36	36	36	36

$^{***}p < 0.01$; $^{**}p < 0.05$; $^{*}p < 0.1$. Standard errors in parentheses.

Table OA.4: OLS regressions of period averages of absolute log market price deviations on absolute log deviations of estimates before information revelation and jar dummies, using only the first periods of trading each jar.

E. Robustness checks

	CDA mean	CDA median	CDA close	CDA mid	CA
Intercept	0.300	0.291	0.401^{**}	0.184	−0.145
	(0.269)	(0.152)	(0.149)	(0.130)	(0.159)
$AbsDev^{pre}$	−0.080	−0.060	−0.299	−0.058	0.530^{**}
	(0.313)	(0.176)	(0.173)	(0.150)	(0.203)
Jar 2	−0.172	−0.184	−0.154	−0.118	0.020
	(0.202)	(0.114)	(0.112)	(0.097)	(0.070)
Jar 3	−0.021	−0.095	−0.039	−0.010	0.001
	(0.197)	(0.111)	(0.109)	(0.095)	(0.070)
R^2	0.152	0.344	0.446	0.273	0.726
Adj. R^2	−0.357	−0.049	0.114	−0.164	0.561
RMSE	0.235	0.133	0.130	0.113	0.075
Num. obs.	9	9	9	9	9

$^{***}p < 0.01$; $^{**}p < 0.05$; $^{*}p < 0.1$. Standard errors in parentheses.

Table OA.5: OLS regressions of period averages of absolute log market price deviations on absolute log deviations of estimates before information revelation and jar dummies, using only the first periods of trading per session.

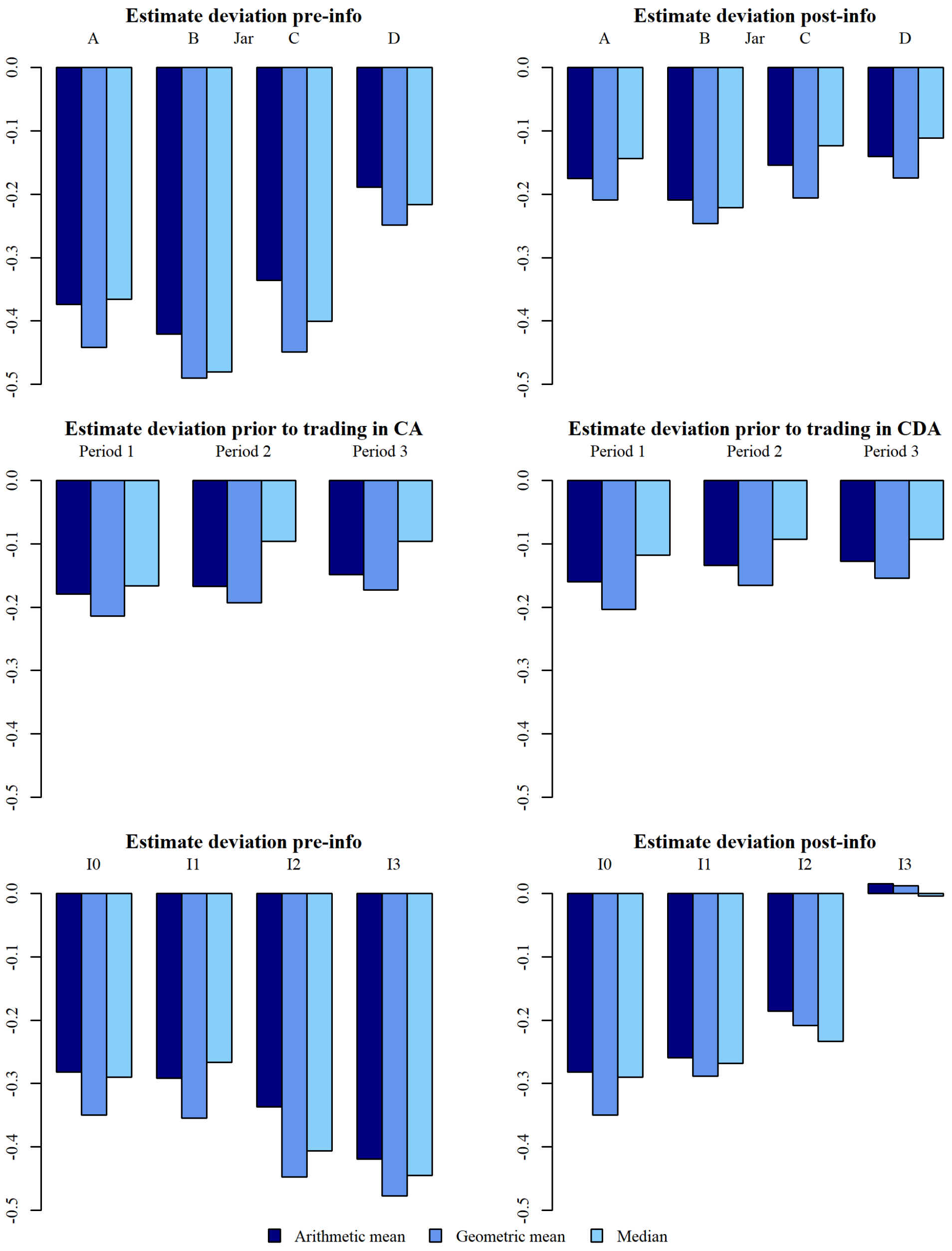

Figure OA.3: Robustness check I: Scatter plots and respective linear fits of average price on different risk measures, using only data from periods 2 through 8. Each small circle represents one period. R^2 stems from linearly regressing average price on the respective risk measure.

F. Experimental instructions

The following is an English translation of the instructions, which were originally provided to participants in German. Red text blocks in square brackets are comments added for the reader and not included in the original instructions. Comments prefaced by ‘CDA:’ constitute parts only included in the instructions for treatment CDA, comments prefaced ‘CA’ constitute parts only included in the instructions for treatment CA.

Dear participants,

welcome to today's experiment.

Please read the instructions for the experiment carefully. Every statement in these instructions is truthful. The size of the payment you will receive at the end of the experiment depends on how well you have understood the instructions. If you have a question, please raise your hand. We will then answer your question in private. The experiment and the analysis of the resulting data will be anonymized.

From now on, please do not speak to other participants and use only those tools the experimenter provides you with. Please turn off all of your electronic devices. At the computer, too, you may only use those tools required for the experiment. If you violate these rules, you will not be paid and will be excluded from future experiments.

Thank you for your attention and for participating in today's experiment.

Structure of the experiment:

This experiment consists of two parts – a market experiment and a closing experiment, which will involve further short decision-making situations. You can earn money in both parts of the experiment. Prior to the experiments, you will be informed about the rules in separate sets of instructions.

Experiment 1: Market experiment

Instructions for the trading screen

Trial period

Instructions for the assets being traded

Experiment

Questionnaire

Experiment 2: Closing experiment

Instructions and experiment

Questionnaire

Payment

[Note to the reader: End of page 1]

(1) INSTRUCTIONS FOR THE TRADING SCREEN:

In this experiment you can buy and sell jars filled with coins (€1, 20, 5, and 1 cents). Following [CA: two trial periods] [CDA: a trial period], you will receive more information about these jars. You will be trading in a [CDA: continuous] double auction. This means that every trader can act as a buyer and as a seller. You can submit as many buy and sell orders [CA: in a period] as you wish by entering a price you are willing to trade at (with a maximum of two decimal places) [CDA: and then clicking "Buy order/Sell order"]. Every order is for exactly 1 jar filled with coins.

[CA: When you are done creating your buy and sell orders, you can send them to the market using the "Send orders" button. The market will determine a uniform price from all traders' orders such that the number of jars bought and sold is equal. If you submit a number of high buy offers, the price will tend to rise, and vice versa. All buy orders with prices higher than the uniform price and all sell orders with prices below the uniform price will be transacted at the uniform price. This means that you will sell as many jars as you have offered to sell below this price and you will buy as many jars as you offered to buy above this price. For every jar you ***buy***, your euro balance will decrease by the price and your balance of jars will increase by 1, and vice versa for every jar you ***sell***.]

[CDA: All orders will be sorted by price, such that the best will be on top and will have a blue background. The best order can be accepted using the buttons "Buy" or "Sell". When you ***buy*** a jar, your euro balance will decrease by the price and your balance of jars will increase by 1. Conversely, when you ***sell*** a jar, your euro balance will increase by the price and your balance of jars will decrease by 1. Please note that you can only buy (sell) as many jars as are covered by your euro balance (jar balance). Negative balances of euros or jars (short sales) thus are not permissible. The computer will delete orders which are no longer covered.]

[Note to the reader: Screenshot CA]

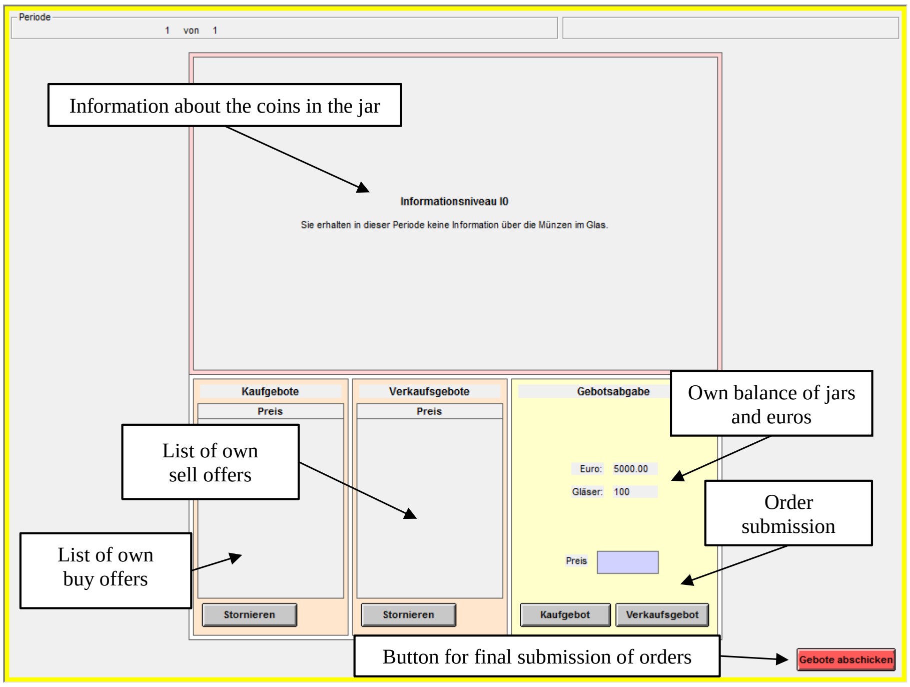

[Note to the reader: Screenshot CDA]

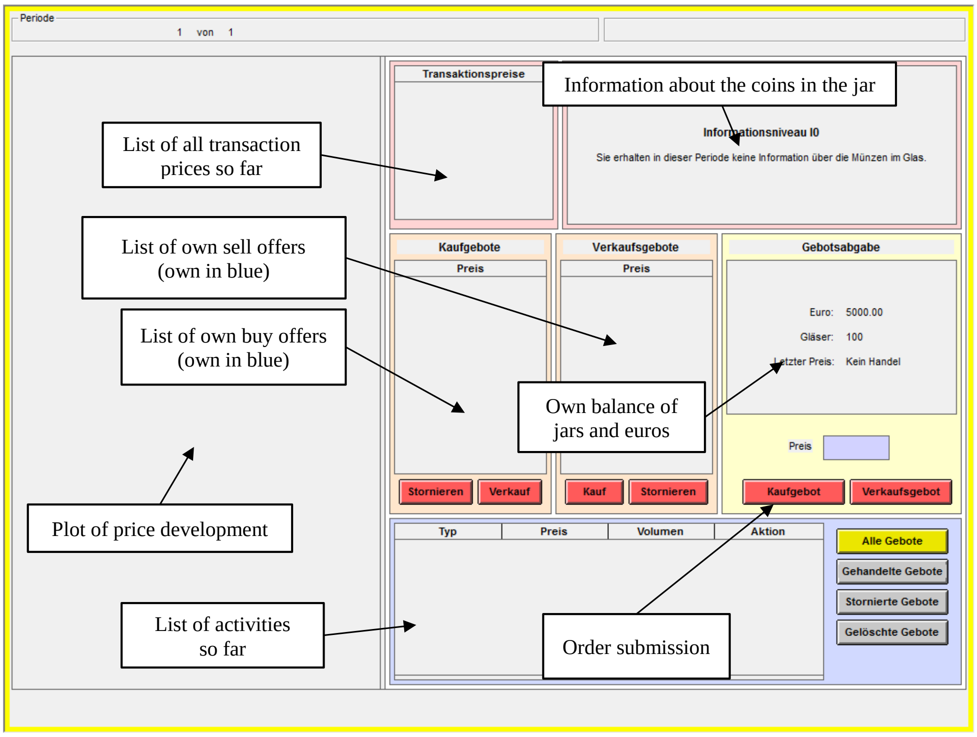

We will now conduct [CA: two trial periods of 2 minutes each] [CDA: a trial period of 5 minutes] to familiarize you with the trading interface. Use these to train the use of the trading system. The trial [CA: periods do] [CDA: period does] not affect your payment. [CA: After every trial period you will see how many glasses you would have bought or sold at different possible prices.]
[Note to the reader: End of page 2]

(2) STRUCTURE OF THE EXPERIMENT:

The experiment is structured into **12 periods** of 3 minutes each. Remember: In this experiment you can buy and sell jars filled with coins (€1, 20, 5, and 1 cents). Every participant starts into every period with an endowment of 5 jars and a certain euro balance. Not every trader will get the same euro balance in every period. The trading prices of the jars are determined by the supply and demand of yourself and the other seven traders in your row (only the eight traders in the same row trade with each other).

[Note to the reader: Subjects were seated in three rows of eight computers each in the Innsbruck EconLab.]

You will be trading each jar (there is a total of 4 different jars, referred to as A, B, C, D) in three consecutive periods and your starting endowment in each period will be 5 jars and a euro amount.

	Periods 1-3	Periods 4-6	Periods 7-9	Periods 10-12
Row 1	Jar type A	Jar type B	Jar type C	Jar type D
Row 2	Jar type B	Jar type C	Jar type D	Jar type A
Row 3	Jar type C	Jar type D	Jar type A	Jar type B
unused	Jar type D	Jar type A	Jar type B	Jar type C

At the start of each 3-period-block, jars of the corresponding type will be handed out to be inspected by the participants (eight participants each receive the same jar and each one can view it, handle it, etc. for 15 seconds). After three trading periods, the same eight people will be handed another jar for inspection. After you have handled the jar, you will be asked to submit, on the computer, an estimate of the total value of all coins in the jar.

After you have submitted this estimate, you will learn from your screen the number and value of different coin types contained in the jar. There is a total of four information levels (I0, I1, I2 and I3). Out of the eight participants in one row, two each will receive the same information for three periods (each market thus twice contains I0, I1, I2 and I3). Depending on your information level, you will learn the number of none, one, or two types of coins contained in the jar. You will also learn the value of these coins. A participant with information level I0 learns nothing about the number of coins; I1 learns the number and value of the 1€ coins; I2 the number and value of the 1€ and 20 cent coins, and I3 (the best informed) the number and value of the 1€, 20 cent and 5 cent coins. Nobody learns the number or value of the 1 cent coins. The information structure looks as follows:

	1€	20 cent	5 cent	1 cent
I0				
I1	known			
I2	known	known		
I3	known	known	known	

[Note to the reader: End of page 3]

After you have received the information, you will again be asked to estimate the value of all coins in the jar. You will also be asked to again estimate the value of the coins in the most recently circulated jar at the beginning of periods where no new jar is handed around. At the end of the experiment, you will be paid 20 cents for every estimate that deviates by a maximum of 5% from the true value of the coins in the jar, 10 cents if the estimate deviates by a maximium of 15%, and 5 cents if the estimate deviates by a maximum of 25%. In total you can earn another 3 euros from your estimates. After submission of the estimates, trading begins.

After three trading periods, participants receive information about the new jar type, and the individual information level changes. Each participant receives each information level once, which means that no one is advantaged or disadvantaged over the total duration of the experiment. The trading phase is identical in each period. You can decide whether you want to sell jars to other participants or whether you want to buy from other participants.

(3) OVERVIEW SCREEN:

After trading has ended, you will see an overview screen which will display your balance of jars and euros. You will start each period anew with 5 jars and a certain euro amount. After 3 consecutive periods you will start with a new jar (A, B, C or D) and a new information level (I0, I1, I2 or I3).

(4) PAYMENT:

After all 12 periods are over, your payment will be calculated based on the jars and euros you hold after trading in one (randomly chosen) period out of the 12 periods. For this, your ending wealth (=euro balance plus value of the coins in the jars) in a randomly chosen period will be taken and divided by 30. This yields the euro amount you will have earned from the trading part of the experiment. Added to this will be the euro amount you will have earned from your estimates of the values of the coins in the jars. You will be paid the sum of these two amounts.

Once again the most important details for the experiment:

- There are 12 trading periods; the same jar will be traded in all periods of a given block of three consecutive periods (a total of four different jars A, B, C and D).
- Every participant starts into each period with 5 jars and a certain euro amount.
- There are four information levels I0, I1, I2 and I3. In each row, two participants each will receive the same information level regarding a jar's contents. Across the 12 periods, every participant will have every information level once, for three periods.
- After the period, every jar pays the exact value of the coins contained in the jar.
- The final payment will be calculated from the true value of the jars and the euro balance in one of the 12 periods (randomly chosen), divided by 30. Added to this will be the payment for the estimates (up to 3 euros).

[Note to the reader: End of page 4]
